# Coming Back to Physiology: Extra Hepatic Functions of Proprotein Convertase Subtilisin/Kexin Type 9

**DOI:** 10.3389/fphys.2020.598649

**Published:** 2020-12-07

**Authors:** Klaus-Dieter Schlüter, Annemarie Wolf, Rolf Schreckenberg

**Affiliations:** Institute of Physiology, Justus-Liebig-University, Gießen, Germany

**Keywords:** NARC1, LDL, inflammation, cholesterol transport, lectin-like oxidized low-density lipoprotein receptor-1

## Abstract

Neuronal apoptosis regulated convertase-1 (NARC-1), now mostly known as proprotein convertase subtilisin/kexin type 9 (PCSK9), has received a lot of attention due to the fact that it is a key regulator of the low-density lipoprotein (LDL) receptor (LDL-R) and is therefore involved in hepatic LDL clearance. Within a few years, therapies targeting PCSK9 have reached clinical practice and they offer an additional tool to reduce blood cholesterol concentrations. However, PCSK9 is almost ubiquitously expressed in the body but has less well-understood functions and target proteins in extra hepatic tissues. As such, PCSK9 is involved in the regulation of neuronal survival and protein degradation, it affects the expression of the epithelial sodium channel (ENaC) in the kidney, it interacts with white blood cells and with cells of the vascular wall, and it modifies contractile activity of cardiomyocytes, and contributes to the regulation of cholesterol uptake in the intestine. Moreover, under stress conditions, signals from the kidney and heart can affect hepatic expression and thereby the plasma concentration of PCSK9 which then in turn can affect other target organs. Therefore, there is an intense relationship between the local (autocrine) and systemic (endocrine) effects of PCSK9. Although, PCSK9 has been recognized as a ubiquitously expressed modifier of cellular function and signaling molecules, its physiological role in different organs is not well-understood. The current review summarizes these findings.

## Introduction

Neuronal apoptosis regulated convertase-1 (NARC-1) was initially identified in cultured cerebellar granule neurons (CGNs) in which NARC-1 messenger RNA (mRNA) was upregulated when cells were exposed to pro-apoptotic stimuli such as the withdrawal of potassium or serum (Chiang et al., 2001). Neuronal apoptosis is required for normal development of the brain but it is also involved in neurodegenerative disorders. The mRNA of NARC-1 encodes a protein that was identified as a novel member of a class of proteinase-K-like serine proteases and is now best known as proprotein convertase subtilisin/kexin type 9 (PCSK9). Therefore, the names NARC-1 and PCSK9 are synonyms for the same protein.

In 2003, PCSK9 was identified as an important regulator of the hepatic low-density lipoprotein (LDL) receptor (LDL-R) membrane abundance (Seidah et al., 2003). Within a few years, there was a large expansion in the field concentrating on the role of PCSK9 in the regulation of LDL-R and subsequently LDL cholesterol (LDL-C). Multiple factors and conditions affect the hepatic expression of PCSK9, i.e., age, sex, pregnancy, diet, and diurnal variation (see Persson et al., 2010; Cui et al., 2015; Saely and Drexel, 2016). Two main transcription factors have also been found to be responsible for PCSK9 expression in hepatocytes, namely sterol response element binding protein (SREB)-2 and hepatocyte nuclear factor (HNF)-1*α* as summarized in detail in earlier studies (Cui et al., 2015; Glerup et al., 2017). In particular, HNF-1*α* has an interesting regulation, as insulin *via* the activation of the mammalian target of the rapamycin (mTOR) pathway leads to the nuclear extrusion of HNF-1α and thereby a reduction in PCSK9 expression. On the other hand, insulin resistance leads to a lack of nuclear HNF-1 extrusion, thereby favoring the excessive release of PCSK9 (reviewed by Lagace, 2014). Collectively, these data identified PCSK9 as a key player in hypercholesterolemia.

Proprotein convertase subtilisin/kexin type 9 was identified as a potential target for the pharmacological treatment of patients who suffer from hypercholesterolemia. Antibodies directed against PCSK9 (alirocumab and evolocumab) were introduced into clinical practice within a few years. Both antibodies are safe, effective, and improve the therapeutic portfolio for patients with hypercholesterolemia especially for patients with statin intolerance (Glerup et al., 2017). Administration of siRNA directed against PCSK9 (inclisiran) may become a therapeutic alternative because this drug is effective and can be administered less frequently than antibodies. However, the success in this field has led to a selective view on PCSK9 as a regulator of hepatic expression in LDL-Rs and thereby in LDL-C. Less attention has been directed to the question of the physiological function of PCSK9 in the whole organism, although it is well accepted that PCSK9 is expressed in nearly all tissues in mammalians (Seidah et al., 2003). Furthermore, PCSK9 knockout mice (PCSK9^−/−^) are hyperglycemic and exhibit pancreatic islet abnormalities (Mbikay et al., 2010). In addition, PCSK9^−/−^ mice develop severe diet-induced non-alcoholic steatohepatitis (Lebeau et al., 2019). These findings already support the significance of PCSK9 in physiological but also pathological processes in extra hepatic tissues. Nevertheless, the levels of PCSK9 expression in extra hepatic tissues may be lower than those reported for the liver (Schlüter et al., 2017). However, a better understanding of the physiological functions of PCSK9 in extra hepatic tissues will help to estimate the potential side effects of targeting PCSK9 but also to identify potential new therapeutic implications, especially in the field of Alzheimer’s disease (AD), inflammation, heart failure, and cancer.

**Figure 1:**
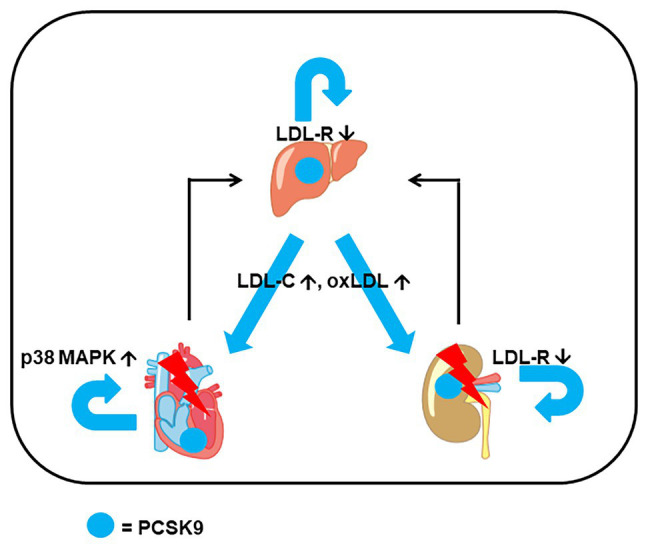
Example for extrahepatic expression of proprotein convertase subtilisin/kexin type 9 (PCSK9) and interaction between organs. In addition to hepatic expression of PCSK9, it is also expressed in heart and kidney. Triggered by stress, heart, and kidney can release PCSK9 and cytokines that affect hepatic expression. The combined effect of locally and systemically increased PCSK9 stresses the heart (*via* p38 MAP kinase activation), the kidney [*via* low-density lipoprotein (LDL) receptor (LDL-R) downregulation and impairment of metabolism] increases the level of LDL cholesterol (LDL-C) and oxidized LDL (oxLDL) *via* hepatic induction of PCSK9 that finally adds additional stress to the heart and kidney.

In general, there are three different issues to address when talking about the extra hepatic effects of PCSK9. First, we need to identify how hepatic-derived PCSK9 affects the physiological function of non-hepatic tissues, i.e., by the identification of potential target molecules of PCSK9. Second, we must understand how extra hepatic-released PCSK9 affects the liver and vice versa and the lipid metabolism in a more general way. Finally, we need to identify the potential roles of endogenously expressed PCSK9 in extra hepatic tissues as a paracrine or autocrine factor (Figure 1). In this review, we summarize articles that were found in the PubMed data bank using the search criteria PCSK9 and kidney, PCSK9 and muscle, PCSK9 and brain, PCSK9 and heart, and PCSK9 and vessels.

## The Role of PCSK9 in the Brain

In neuronal studies, the name NARC-1 is often used instead of PCSK9. This directs attention to the foremost identified correlation between the induction of NARC-1 mRNA expression and neuronal apoptosis. Neuronal apoptosis is required for normal development. Evidence that NARC-1/PCSK9 is important in neuronal apoptosis is supported by the quantification of PCSK9 expression in different regions of the brain. Local expression of PCSK9 in the anterior lobules of the cerebellum is approximately 8-fold stronger than in posterior lobule X that is resistant to neurodegeneration (Martin et al., 2020). In Zebrafish, silencing PCSK9 resulted in general disorganization of cerebellar neurons (Poirier et al., 2006). Similarly, when inducing neuronal differentiation in P19 cells by retinoic acid, NARC-1/PCSK9 is upregulated within 2 days (Poirier et al., 2006). Furthermore, serum levels of PCSK9 are lower in the sera, spinal cords, and placenta of rats with spina bifida aperta when experimentally induced by all-trans retinoic acid (An et al., 2015). All these findings suggest that PCSK9 plays a critical role in at least the early phases of neuronal differentiation. In mice with a high intrinsic motivation to run, PCSK9 expression in the cerebellum was significantly lower suggesting participation in the behavior of mammalians (Zhang et al., 2018). However, PCSK9^−/−^ mice do not show clear signs of neurodegeneration. Similarly, loss-of-function carriers do not show obvious defects in neuronal development (O’Connel and Lohoff, 2020). Thus, the role of PCSK9 in brain development may be different between mammalians and fish, and this topic requires future research for clarification.

When NARC-1/PCSK9 is overexpressed in CGNs, these cells develop more signs of apoptotic cell death than those with physiological levels of NARC-1 (Bingham et al., 2006). Similarly, PCSK9 promotes apoptosis in PC12 cells exposed to oxidized LDL (oxLDL; Liu et al., 2017). However, the mechanism by which NARC-1/PCSK9 induces neuronal apoptosis remains elusive (reviewed in more detail by O’Connel and Lohoff, 2020). Obviously, the effect can only in part be linked to caspase activation, a classical pro-apoptotic pathway. In neurons, LDL-R expression is regulated by the Myosin regulatory light chain-interacting protein/inducible degrader (Mylip/Idol) of the LDL-R rather than by PCSK9, whereas that of the very low density lipid (VLDL) receptor depends on the Hypoxia-inducible factor 1*α* (HIF1*α*)/Wnt pathway (Do et al., 2016; Kysenius and Huttunen, 2016). Nevertheless, LDL-R expression in neurons is low (Poirier et al., 2006). As neuronal apoptosis in Niemann-Pick disease is associated with abnormal cholesterol storage and although neuronal expression of LDL-Rs is low, the hypothesis that neuronally expressed VLDL receptors and ApoER2 are potential targets of PCSK9 was tested. PCSK9 may trigger apoptosis *via* reelin, a natural ligand of these molecules with a signaling character but experiments with double knockout mice (PCSK9^−/−^ and reelin^−/−^) did not support this hypothesis. In the case of the VLDL receptor, PCSK9 is not involved in its regulation (Kysenius and Huttunen, 2016). However, PCSK9 may trigger apoptosis *via* ApoER2 but the subsequent intracellular pathways are not yet identified (Kysenius et al., 2012; Wang et al., 2018). Thus, it seems that PCSK9 target molecules differ between the brain and liver.

Proprotein convertase subtilisin/kexin type 9 (mRNA and protein) expression is elevated in the frontal cortices of AD patients together with other biomarkers of AD suggesting a role for PCSK9 in disease progression (Picard et al., 2019). When mechanistic insights related to AD were analyzed in more depth, *β*-site amyloid precursor protein-cleaving enzyme-1 (BACE-1) was identified as a potential target of PCSK9 in neurons. BACE-1 undergoes acetylation in the ER/Golgi compartment and its translocation to the cell membrane seems to be required for the deposition of amyloid β(Aβ)-peptide, a characteristic feature of AD (Ko and Puglielli, 2009). Data on PCSK9 silencing experiments suggest that PCSK9 is involved in a process that regulates BACE-1 expression. Consequently, PCSK9^−/−^ mice have increased BACE-1 and increased Aβ in the brain (Jonas et al., 2008). In PC12 cells, silencing PCSK9 could also increase the amount of Aβs (Liu et al., 2017). In contrast, ApoE^−/−^ mice, receiving a high-fat diet, had an increased cerebellar expression of PCSK9 and BACE-1 (Zhao et al., 2017). A mechanistic explanation for this unexpected result may come from studies by which ceramide was used to stress neurons. In such experiments, an increased expression of two acetyltransferases protects BACE-1 from PCSK9-dependent downregulation (Ko and Puglielli, 2009). In line with these studies, the use of PCSK9 inhibitors was associated with a moderately increased risk of AD in an AD risk patient collective but not associated with the general development of cognitive dysfunction (Giugliano et al., 2017; Williams et al., 2020). To what extent PCSK9 regulates neuronal apoptosis under physiological or pathophysiological conditions is still a matter of debate. Despite the aforementioned discrepancy between apoptosis, neuronal development, and lack of effect of PCSK9 deficiency in mice, it must be noted that oxidative stress in the brain *via* oxLDL increases PCSK9 expression and PCSK9-dependent apoptosis *via* the classical bcl-2/bax caspase3/9 pathway, whereas at the same time its effect on BACE-1 reduces the appearance of Aβ and Aβ-dependent apoptosis (Wu et al., 2014). Although all the aforementioned studies linked PCSK9 expression with the control of BACE-1-dependent Aβ deposition, published studies did not reveal a clear picture about the role of PCSK9 and AD. The main arguments against a role of PCSK9 in AD come from the following studies. First, loss-of-function mutations of PCSK9 are not related to neurocognitive deficiency (Mefford et al., 2018; Paquette et al., 2018). Second, the concentration of PCSK9 in the cerebrospinal fluid does not correlate with AD (Courtemanche et al., 2018). Third, genetic manipulation of PCSK9 in mice does not affect BACE-1 expression suggesting that the aforementioned opposite results are not consistent in all mice strains (Liu et al., 2010). Nevertheless, lowering LDL-C reduces AD risk (Benn et al., 2017). One alternative possibility on how LDL-C, PCSK9, and AD are linked to each other may be that PCSK9 contributes to vascular stress (see below) that then affects the blood-brain barrier and favors inflammation (Zhao et al., 2016). Other potential PCSK9 target molecules that may affect AD are VLDL receptors and CD36 (Oldham et al., 2018). However, as the neuronal expressions of them are not affected by PCSK9 deficiency in mice such an interaction remains unclear at the moment. The most likely explanation for correlations between hypercholesterolemia and AD is that hepatic expression of PCSK9 and subsequently of receptors such as VLDL and LDL-related protein-1 (LRP-1) is affecting the brain in a more indirect way. There is little consensus about the brain-specific effects of PCSK9 and its role in AD or neuronal development regardless of very interesting experimental data.

Finally, it was investigated whether PCSK9 plays a role in stroke. Data investigating loss-of-function carriers showed an association between chronic heart disease and myocardial infarction but not stroke (Kent et al., 2017; Hopewell et al., 2018).

In summary, PCSK9 is constitutively expressed in various regions of the brain, where it is linked to apoptosis and degradation of BACE-1. BACE-1 may be linked to A*β* deposition and AD, a process that may depend on PCSK9. This interaction has been reviewed previously in more detail (Adorni et al., 2019). High expression of PCSK9 is found under conditions of low voluntary running motivation, unfavorable diet, and oxidative stress. In contrast, its role in neuronal differentiation remains unclear, especially in mammalians.

## The Role of PCSK9 in the Kidney

The kidney was among the first tissues known to express PCSK9 (Seidah et al., 2003). Detailed *in vitro* studies using HEK 293 cells as a model indicated that PCSK9 targets three subunits of the epithelial sodium channel (ENaC; Sharotri et al., 2012). The dysfunction of ENaC has been attributed to altered sodium reabsorption in the terminal part of the nephron leading to alteration in blood pressure. Therefore, it was expected that a deficiency in PCSK9 *in vivo* would either induce dysregulation in sodium plasma concentration or change blood pressure. However, in PCSK9^−/−^ mice, the absence of PCSK9 did not affect either of these two parameters, neither under basal nor under stress conditions (Berger et al., 2015). As PCSK9 regulates the hepatic expression of LDL-Rs, and LDL-Rs contribute to the clearance of lipopolysaccharides (LPS) from circulation, PCSK9 deficiency was expected to improve the outcome in animal sepsis models including sepsis-dependent renal dysfunction. That is indeed the case (Dwivedi et al., 2016). Furthermore, LPS induces the renal and hepatic expression of PCSK9, thereby reducing the ability of LDL-Rs to remove LPS from circulation (Feingold et al., 2008). However, the translation of such experimental data on human pathophysiology is not guaranteed, since PCSK9 does not seem to be relevant in inflammatory processes in humans (Heinzl et al., 2020).

Irrespective of chronic inflammation, renal expression and secretion of PCSK9 in circulation is further induced by local and tissue-specific damage, such as podocyte damage induced by factors from nephrotoxic serum or hypercholesterolemia (Haas et al., 2016). Of note, renal disease can affect the hepatic expression of PCSK9 (Liu and Vaziri, 2014; Sucajtys-Szulc et al., 2016a,b). Mechanistically, it was proposed that damaged podocytes cause an activation of tumor necrosis factor (TNF)-*α* that triggers the hepatic upregulation of PCSK9 (Busuioc et al., 2019). As hepatic-derived PCSK9 accounts for the majority of circulating PCSK9, this can affect the expression of target proteins in the kidney as well, creating a feedback loop by which the kidney under stress conditions adapts its own function *via* hepatic-derived PCSK9. At least, hepatic-derived PCSK9 can downregulate the renal expression of LDL-Rs (Schmidt et al., 2008). As expected from this assumption, targeting plasma PCSK9 in hypercholesteremic mice attenuated LDL-R expression in the kidney and subsequently renal lipid accumulation (Wu et al., 2020). The latter was associated with renal fibrosis. Using an inhibitor of intestine cholesterol adsorption (ezetimibe), it was shown that hepatic and renal expression of PCSK9 increased as a part of a compensatory mechanism (Xu et al., 2015). On the other hand, in an adriamycin-induced nephropathy model, renal expression of PCSK9 decreased as well as that of its transcriptional activator HNF-1α, whereas that of LDL-Rs increased and lipid deposition in renal cells was favored (Zhang and Li, 2019). Moreover, the abovementioned triggering of hepatic PCSK9 expression in nephrotic syndrome increased the level of LDL-C that by itself acts as a risk factor for kidney function (Busuioc et al., 2019). Therefore, it is important to note that PCSK9 inhibition by administration of antibodies such as evolocumab is efficient in patients with different degrees of chronic kidney disease (Charytan et al., 2019; Lee et al., 2019).

In conclusion, there is good evidence that an intense cross-talk between the liver and kidney exists, that specifically regulates the renal expression of LDL-Rs. Renal downregulation of LDL-Rs protects the kidney from lipid storage and subsequent induction of renal fibrosis. It seems that under certain conditions, the kidney can transmit signals to the liver that increase the secretion of PCSK9 from the liver that then affects and reduces the expression of LDL-Rs in the kidney. In contrast, although initially considered to be important, based on experiments with HEK 293 cells, ENaC seems not to be a major target for PCSK9 in the kidney or does PCSK9 directly affect blood pressure.

## The Role of PCSK9 in the Vasculature

The vasculature is the barrier between the blood and tissue. As PCSK9 is secreted into the blood, the endothelial cells are the first-line target cells for PCSK9 at this barrier. Nevertheless, other cells of the vascular wall, such as smooth muscle cells, are also potential target cells of PCSK9. The physiological and potential pathophysiological relationship between the vasculature and PCSK9 goes far above this direct interaction. Cells of the vasculature, namely endothelial and smooth muscle cells, express PCSK9 itself. Smooth muscle cells express more PCSK9 than endothelial cells (Ding et al., 2015). Smooth muscle cells with a lower expression of PCSK9 are in a more differentiated state and less proliferative. This supports wound healing (Ferri et al., 2016). In aortic smooth muscle cells, angiotensin II (Ang II) can reduce the expression of PCSK9 and thereby increases the number of LDL-Rs on the membrane (Sakamoto et al., 2015). Of note, there is little evidence for a direct interaction between Ang II and PCSK9, but high levels of cholesterol can modify the activity of Ang II (Lu et al., 2016). There are several targets for PCSK9 in different vascular cells as summarized in an earlier study (Karagiannis et al., 2018).

Proprotein convertase subtilisin/kexin type 9 was identified as a potential target of miR-17-5p in atherosclerotic plaques (Tan et al., 2017). Moreover, within the blood, circulating inflammatory cells express PCSK9 and are targeted by PCSK9. PCSK9 can alter their function, too. *Via* this pathway, PCSK9 does not only directly affect the cells of the vasculature but also in an indirect way, as PCSK9 switches white blood cells into a pro-inflammatory state releasing pro-inflammatory cytokines. Finally, PCSK9 can directly affect the formation of inflammatory cells by modifying T cell programming (Kim et al., 2019). In general, it is accepted that high levels of PCSK9 are associated with pro-atherosclerotic conditions (Sithu et al., 2017). Therefore, the relationship between PCSK9 and the vasculature is the subject of intensive research. When targeting plasma PCSK9 by the administration of antibodies, plaque size is reduced in relation to the lowered LDL-C but the phenotypical characterization of the plaque is not changed (Nicholls et al., 2018). This suggests that the aforementioned effects of PCSK9 on vascular composition are due to the autocrine and paracrine effects of PCSK9. In line with these suggestions that PCSK9 exerts LDL-R-independent effects related to atherosclerosis, a triple knockout mouse with PCSK9^−/−^, LDL-R^−/−^, and ApoB^−/−^ revealed less atherosclerosis than an LDL-R^−/−^ and ApoB^−/−^ double knockout mouse without any differences in cholesterol levels (Sun et al., 2018). Nevertheless, PCSK9 is mainly a modifier of atherosclerosis rather than a trigger itself. When a gain-of-function mutation of PCSK9 was administered into pigs, molecular adaptation was clearly seen but a subsequent physiological measurable difference with endothelial dysfunction required the administration of an atherogenic diet (Hedayat et al., 2018).

Endothelial cells also express PCSK9. As in hepatocytes, PCSK9 directly controls the level of LDL-R expression in endothelial cells (Milasan et al., 2016). Moderate shear stress (3–6 dynes/cm^2^) induces reactive oxygen species (ROS) that trigger the expression of PCSK9 in endothelial cells (Ding et al., 2015). PCSK9 itself can increase the endothelial expression of oxLDL receptors (LOX-1); thereby favoring the unrestricted uptake of cholesterol by these cells (Ding et al., 2015). Furthermore, PCSK9 amplifies LOX-1-dependent apoptosis in endothelial cells, *via* the activation of stress-dependent MAP kinase pathways (p38) and subsequently triggering apoptosis *via* bcl-2/bax and caspase 3 (Li et al., 2017). This process can be further improved by pro-inflammatory stimuli such as LPS [*via* toll-like receptor-4 (TLR-4)] or oxLDL (Ding et al., 2015, 2016; Li et al., 2017). A similar mechanism was also described for smooth muscle cells in the same publication. It seems that pro-inflammatory processes increase mitochondrial-derived ROS. Within the vasculature, aortic branching and aorta-iliac bifurcation are not only regions of low shear stress but also oxidative stress. At these areas, low shear stress produces a self-reinforcing upward spiral by which oxidative stress triggers the expression of PCSK9 that then initiates the increased expression of LOX-1, which finally supports the formation of atherosclerotic plaques.

Within a pro-inflammatory process, the activation of PCSK9 expression and secretion from smooth muscle cells into the blood stream can downregulate LDL-Rs in monocytes. Subsequently, monocyte cholesterol uptake is reduced from what is normally required to induce the expression of C-C chemokine receptor type 2 (CCR2) on monocytes. This avoids monocyte migration (Grune et al., 2017). Macrophages, as monocytes are named once they have moved from the blood into a tissue *via* a process called diapedesis, play an important role in the development of atherosclerotic plaques. Excessive cholesterol uptake transforms macrophages into foam cells that are typical for atherosclerotic plaques. Cholesterol efflux from macrophages, however, prevents the formation of foam cells in the vascular wall and is therefore anti-atherosclerotic. This process is attenuated by PCSK9 (Shen et al., 2013). Cholesterol efflux requires a sufficient expression of ATP binding cassette transporter A1 (ABCA1). PCSK9 reduces the expression of ABCA1 and thereby favors the pro-atherosclerotic status of macrophages within the vascular wall (Adorni et al., 2019). PCSK9 may be directly secreted by adjacent smooth muscle cells (Ferri et al., 2012; Adorni et al., 2017). Furthermore, PCSK9 seems to be required to induce fatty acid translocase (FAT = CD36) and LOX-1 in macrophages whereas that of LDL-R is reduced (Tang et al., 2010; Ferri et al., 2012; Ding et al., 2018a). In the liver, however, PCSK9 reduces the expression of hepatic CD36 (Lebeau et al., 2019). Nevertheless, PCSK9-dependent induction of CD36 and reduction of ABCA1 in macrophages supports foam cell formation in the vasculature. As in other cells as well, PCSK9 can stimulate the production and secretion of pro-inflammatory cytokines from macrophages, a process dependent on LDL-Rs (Ricci et al., 2018). The pro-inflammatory effect of PCSK9 on macrophages seems to be LDL-C independent (Tang et al., 2017). The risk of plaque rupture is increased by the level of calcification of the atherosclerotic plaque. Additional evidence suggested that PCSK9 can also favor plaque calcification (Goettsch et al., 2016; Iida et al., 2018). Macrophages participate in high fat diet-induced local inflammation in multiple organs, such as the liver, kidney, and small intestine (Ding et al., 2020). High-fat diet induces the expression of NOD-, LRR-, and pyrin domain-containing (NLRP)-3 inflammasome that then enables IL-1β secretion. IL-1β triggers the induction of PCKS9 in macrophages (Ding et al., 2020). In NLRP-3 knockout mice, a lower expression of PCSK9 in macrophages was found (Ding et al., 2020). PCSK9 secreted by macrophages can act on cells *via* the induction of TLR-4 (Ding et al., 2020; Badimon et al., 2020). Noteworthy, the secretion of PCSK9 from these cells requires LDL-related protein type 5 (LRP5; Badimon et al., 2020).

In addition to vascular-specific changes in PCSK9 and their direct contribution to pro-inflammatory processes in the development of atherosclerosis, the indirect effects of PCSK9 in chronic inflammatory processes are also discussed. In this aspect, the general assumption is that PCSK9 downregulates the hepatic expression of LDL-R, which is required for the clearance of pro-inflammatory stimuli like LPS (Walley et al., 2014). The assumption is motivated by studies with septic patients showing that carriers with loss-of-function mutations of PCSK9 have a reduced mortality (Genga et al., 2018). However, there is no general effect of PCSK9 or its inhibition on inflammatory markers (Ruscica et al., 2018). In addition, PCSK9 gene variants have been associated with stroke that may either be linked to PCSK9-dependent vascular inflammation or LDL-C (Abbou et al., 2007; Han et al., 2014).

Platelets are also directly exposed to PCSK9 in the blood. They express CD36, a potential target of PCSK9. PCSK9 binding to CD36 in platelets seems to induce oxidative stress and activates platelets (Cammisotto et al., 2020). PCSK9 deficiency conversely reduces platelet activity (Paciullo et al., 2019). As expected, PCSK9 deficiency improves the outcome of venous thrombosis (Wang et al., 2017). The antithrombotic drug ticagrelor reduces the oxLDL-dependent induction of PCSK9 in endothelial cells and attenuates endothelial cell apoptosis (Xia et al., 2019). In addition, platelets are affected by oxLDL, which is indirectly linked to PCSK9 as higher LDL-C levels go alongside with more oxLDL. oxLDL stimulates LOX-1 on platelets that support the PCKS9/CD36-dependent activation of p38 MAP kinase and platelet activation (Gurbel et al., 2017). Plasma PCSK9 is also associated with urinary secretion of thromboxane metabolism again underlining the association between PCSK9 and platelet activity (Pastori et al., 2017).

In summary, PCSK9 and the induction of vascular expression of PCSK9 is causally involved in several steps leading to atherosclerosis, mainly by the downregulation of LDL-Rs and ABCA1 as well as the upregulation of CD36 favoring foam cell formation (Table 1). In addition, PCSK9 favors the secretion of pro-inflammatory cytokines and oxidative stress. Of note, these effects of PCSK9 often occur independently of changes in plasma LDL-C.

**Table 1:** Overview about tissue effects and targets of PCSK9.

Organ	Biological effect/target	Disease
Brain	Apoptosis	Neuronal differentiation
	BACE-1	Alzheimer disease
Kidney	LDL-R, lipid storage	Renal fibrosis
Blood vessels	LDL-R, cholesterol uptake	Atherosclerosis
	ABCA-1, reverse cholesterol transport	Atherosclerosis
	CD36, platelet activity	Thrombosis
Heart	Muscle contractility	Heart failure
Gastrointestinal tract	LDL-R, ApoB, TICE	not known^*^
Pancreas	LDL-R	Diabetes
Fat tissue	VLDL-R, fat storage	not known^*^
Skin	Cell cycling, apoptosis	Psioriasis
Bone	TRAF2, osteogenesis	not known
Ovaria	LDL-R	Polycystic ovary syndrome

* not specifically worked out but likely contribute to hypercholesterolemia.

## The Role of PCSK9 in Striated Muscles

Proprotein convertase subtilisin/kexin type 9 is constitutively expressed in all striated muscles (skeletal muscles and heart muscles). Cardiomyocytes differ significantly from hepatic cells because their cholesterol uptake is low and largely independent from LDL-Rs (Reboulleau et al., 2012). The stimulation of LDL-R subtypes and specifically of LOX-1, results in the induction of PCSK9 expression in cardiomyocytes (Schlüter et al., 2017). It has been described before that LOX-1 interacts with the Ang II type 1 receptor (Yamamoto et al., 2015). Both receptors (LOX-1 and AT-1R) share transduction pathways such as p38 MAP kinase (Mufti et al., 2008; Schlüter et al., 2017). Thus, this suggests that PCSK9 interferes with cardiac function as described for isolated and cultured cardiomyocytes (Schlüter et al., 2017). However, the cellular target of PCSK9 that links the induction of expression with decreased cell function remains to be identified. Whether PCSK9 targets intracellular proteins or acts *via* secretion in cardiomyocytes has been analyzed in detail. In summary, the secretion of PCSK9 by cardiomyocytes *via* Surf-4 is responsible for the effect of oxLDL on cardiomyocytes (Wolf et al., 2020). In line with this, plasma concentration of PCSK9 is associated with heart failure (Chandrakala et al., 2012). This suggests that extracellular PCSK9 mediates the effect. Furthermore, ischemia/reperfusion triggers the cardiac and hepatic release of PCSK9 as well as its cardiac expression (Chandrakala et al., 2012; Zhang et al., 2014; Minana et al., 2020). HIF links ischemia to the upregulation of PCSK9 (Ding et al., 2018b). The inhibition of PCSK9 can improve post-ischemic recovery when applied prior to ischemia but not when applied during reperfusion (Ding et al., 2018b; Apaijai et al., 2019; Palee et al., 2019). This suggests that PCSK9 exerts detrimental effects during ischemia. Altogether, these examples show that PCSK9 is induced under stress conditions in cardiac cells.

Similarly, PCSK9 is also expressed in skeletal muscle where it may also target intracellular proteins that have yet not been identified. However, clinical studies indicate that PCSK9 inhibition has fewer side effects on muscles than other LDL-C-lowering pharmaceuticals, such as statins or ezetimibe (Cheng et al., 2016; Nissen et al., 2016). This suggests that it is not LDL-C but extracellular PCSK9 that negatively affects skeletal muscles as well as cardiomyocytes. In this context, a couple of studies have investigated whether exercise affects the plasma concentration of PCSK9. Completely divergent results were obtained in this context (see below).

In conclusion, PCSK9 is not only constitutively expressed in striated muscles but also its expression is also significantly altered under stress conditions. The main difficulties that have arisen are the identification of target proteins in muscles as well as the inconsistency of data in different exercise studies. Thus, the exact function of muscular PCSK9 is not clear.

## The Role of PCSK9 in the Gastrointestinal Tract

Transintestinal cholesterol excretion (TICE) is a constitutive active pathway for cholesterol elimination that represents an alternative pathway to the classical hepatobiliary pathway. In mice, TICE contributes to approximately one third of the total fecal sterol loss (van der Ven et al., 2009). The molecular mechanisms building up this alternative pathway are not well-understood but require either LDL-Rs or apical multidrug transporter ATP-binding cassette transporter B1 (ABCB1) a and b (Le May et al., 2013). Using PCSK9^−/−^ mice, it was clearly shown that PCSK9 reduces the membrane availability of LDL-Rs in intestinal preparations *via* a mechanism that is most likely similar to that described for the liver. In contrast to the liver, a lack of LDL-Rs can be bypassed by alternative pathways that seem to not be sensitive to PCSK9.

Enterocytes are at the border between nutrients up-taken by the organism and the individual cells and organs of the body. In CaCo-2 cells, a human enterocyte cell line, PCSK9 increases the cellular and secreted level of triglyceride-rich apolipoproteins B (apo B; Le May et al., 2009). PCSK9 decreased LDL-R levels in these enterocytes but increased the apo B mRNA expression and stability as well as the levels of lipid-generating enzymes, such as fatty acid synthase, stearoyl-CoA desaturase, and diglyceride acyltransferase 2 (Rashid et al., 2014). Additionally, PCSK9 promotes the interaction between apo B48 and the triglyceride transfer protein (MTP) during the adsorption of lipids (Rashid et al., 2014). Deficiency conversely leads to fewer apo B, larger triglyceride rich proteins, and shows accelerated uptake in the liver (Le May et al., 2009). This leads to the conclusion that PCSK9 modifies cholesterol absorption and the level of PCSK9 is indeed associated with the concentration of sterol absorption markers. PCSK9 inhibition can reduce this absorption (Brandt et al., 2019). However, the exact role of PCSK9 in cholesterol uptake has still to be defined.

Postprandial lipemia in mice depends on circulating not regional PCSK9 expression in the intestine (Garcon et al., 2020). Furthermore, PCSK9 concentrations are stable during an acute fat load and increase only under a long-time high-fat diet (Cariou et al., 2013). Finally, PCSK9 is not involved in chylomicron retention disease (Georges et al., 2011).

Little is known about the regulation of the expression of PCSK9 in the intestine. However, when rats were fed with an inhibitor of cholesterol adsorption (ezetimibe), the expression of PCSK9 in the intestine increased as well as that of sterol regulatory element-binding protein 2 (SREBP2; Xu et al., 2015). This may be considered as a compensatory mechanism to maintain high cholesterol uptake.

In summary, PCSK9 controls the level of expression of LDL-Rs in enterocytes, thereby affecting cholesterol excretion and adsorption. In contrast to the liver, alternative pathways can bypass these functions independently of PCSK9.

## The Role of PCSK9 in Other Organs

Deficiency of PCSK9 (by a loss-of-function mutation or genetic ablation) generates a phenotype with low LDL-C but high plasma glucose (Mbikay et al., 2010). It is therefore associated with an increased risk of type 2 diabetes mellitus (Mbikay et al., 2015). In the absence of PCSK9, pancreatic islets showed higher intracellular accumulation of cholesterol esters, increased intracellular insulin levels, and a pro-inflammatory state (Mbikay et al., 2010; Da Dalt et al., 2019). This phenotype depends on LDL-Rs. Moreover, locally produced PCSK9 rather than circulating PCSK9, affects the function of the pancreas. In contrast to these experimental data, studies on patients with gastric bypass surgery show a decrease in circulating PCSK9 and an improvement in glucose metabolism that was independent of LDL-Rs (Blanchard et al., 2020). Although both studies confirm a relevant role of PCSK9 for glucose metabolism, the physiological and pathophysiological understanding is incomplete. Nevertheless, it is important to recognize that PCSK9 is constitutively expressed in the pancreas where it affects glucose metabolism.

As in other relevant metabolic cells, PCSK9 is also expressed in adipocytes. In these cells, PCSK9 interacts with CD36 leading to increased levels of CD36 in adipocytes in the case of PCSK9 deficiency (Demers et al., 2015). In the liver, PCSK9 can also directly affect the expression of CD36 (Lebeau et al., 2019). In adipocytes, PCSK9 is considered to act mainly on VLDL-Rs rather than LDL-Rs (Roubtsova et al., 2011). Following this argumentation, PCSK9 increases the level of circulating LDL-C by regulating hepatic LDL-R degradation but limits visceral adipogenesis *via* VDLD-R regulation. Subsequently, PCSK9^−/−^ mice accumulate much more visceral adipose tissue than wild-type mice. As suggested, low levels of PCSK9 favor the expression of LDL-R on adipocytes leading to the excessive up-take of LDL-C. LDL-C then triggers the NLRP-3 inflammasome in adipocytes leading to dysfunctional adipose tissue. This mechanism has been linked to type 2 diabetes (Faraj, 2020). Similarly, fasting reduces plasma PCSK9 and thereby increases LDL-R expression in adipocytes leading to NLRP-3 activation (Cry et al., 2020).

Although the role of PCSK9 in systemic inflammation is still under debate, increased levels of PCSK9 are often observed in cells exposed to pro-inflammatory cytokines. NF-κB and TNF-*α* are involved in psoriasis and atherosclerosis. As mentioned above, PCSK9 supports the formation of atherosclerotic plaques. Therefore, it may also be induced in keratinocytes under pro-inflammatory conditions. Experimental data with keratinocytes confirmed this hypothesis (Luan et al., 2019). Moreover, PCSK9 affects the cell cycle and apoptosis of keratinocytes and may become a target in the context of psoriasis in the future. Indeed, plasma PCSK9 concentration is increased in patients with psoriasis (Krahel et al., 2020a). Moreover, the local expression of PCSK9 is increased in response to local inflammation triggered by agonists of TLR4, LOX-1, or TNFα (Krahel et al., 2020b). More importantly, PCSK9 associated with psoriasis appears to be independent of cholesterol metabolism (Garshick et al., 2020).

Physiological levels of PCSK9 seem to be required for regular periodontal bone regeneration. In periodontal ligament stem cells, PCSK9 suppresses the expression of tumor necrosis factor receptor-associated factor 2 (TRAF2). Thereby, PCSK9 suppresses the effect of TNF-α on NF-κB in these cells and improves osteogenic differentiation (Sun et al., 2018).

Finally, high levels of PCSK9 were found in the ovaria of mice with polycystic ovary syndrome. In these cells, PCSK9 once again regulates the expression of LDL-Rs. In contrast to other tissues, systemic and local PCSK9 seemed to contribute similarly to this regulation (Wang et al., 2019).

In summary, the expression of PCSK9 can be detected in nearly all cells. In some of them, PCSK9 targets specific proteins such as TRAF2 or VLDL-Rs rather than LDL-Rs (Table 2).

**Table 2:** PCSK9 and its interaction with the vasculature.

A) Vascular cells		
	PCSK9	Effect of PCSK9
Endothelial cells	+	PCSK9 →LDL-R, LOX-1 ↑
Smooth muscle cells	++	Ang II → PCSK9 ↓ → LDL-R ↑
Macrophages		PCSK9 → ABCA1 ↓ → Cholesterol Efflux ↓
		PCSK9→ CD36 ↑
B) Cells interacting with the vasculature		
Leukocytes	+	PCSK9 → Pro-inflammatory cytokines ↑
Monocytes		PCSK9 → LDL-R ↓ → CCR2 ↓ → Migration ↓
Platelets		PCSK9/CD36 → Thrombosis ↑

→, effect on; ↓, reduction; ↑, induction; LDL-R, low density lipoprotein-receptor; LOX-1, ox-LDL receptor; Ang II, angiotensin II; ABCA1, ATP binding cassette transporter A1; CCR2, C-C- chemokine receptor type 2; and CD36, fatty acid translocase (FAT = cluster of differentiation 36).

## Effects of Exercise on PCSK9 Expression and Function in Different Organs

The aforementioned ubiquitous expression of PCSK9, its significant role in the regulation of metabolism, and its high plasma levels in combination with hypercholesterolemia and inflammation make PCSK9 an attractive target in various settings. This has led to the question of whether the beneficial effects of high physical activity *via* metabolic effects can be explained by side effects of PCSK9 expression and secretion. The findings are, however, not clear. In patients with coronary artery disease, PCSK9 levels increased with improvement in fitness and visceral fat mobilization (Boyer et al., 2016). Similarly, PCSK9 plasma levels increased in participants with common risk factors such as obesity or hypertension although LDL-C decreased (Sponder et al., 2017). In contrast, in healthy subjects, increased physical activity was associated with a decrease in PCSK9 plasma levels (Kamani et al., 2015). In an experimental setting, ovariectomy of female rats reduced the hepatic expression of PCSK9 but exercise had no effect on the changed expression (Sock et al., 2016). In contrast, intestinal expression of PCSK9 was upregulated by voluntary exercise in rats (Farahnak et al., 2018). The authors suggest that this is linked to improved TICE. In mice undergoing a high-fat diet, exercise increased the hepatic expression of SREBP2 and PCSK9 but reduced PCSK9 plasma concentration (Wen et al., 2013). As reviewed previously, exercise has no clear connection to PCSK9 (Saely and Drexel, 2016). Another link between exercise and PCSK9 comes from the observation that exercise increases the secretion of fibroblast growth factor 21 (FGF21), a myokine that affects adipocytes releasing adiponectin. FGF21 reduces the expression of SREBP-2, a transactivator of PCSK9 (Huang et al., 2017).

In summary, in most studies dealing with the interaction between PCSK9 and exercise, PCSK9 is associated with increases in either the cellular expression level or plasma concentration but this effect is completely dissociated from the beneficial effects in plasma LDL-C. The underlying mechanisms are poorly understood.

## PCSK9 and Cancer and What can be Learned From This for Physiological Functions of PCSK9 in Extra Hepatic Tissues

The interest in studies connecting PCSK9 expression in extra hepatic tissues with the progression of cancer comes from the initial findings that PCSK9 can induce apoptosis in the brain. Furthermore, as mentioned before in several chapters, PCSK9 can sensitize stress pathways leading to apoptosis as well. In the context of cancer, however, there are no strong data supporting the concept that PCSK9 in general affects cancer incidence. Studies with loss-of-function carriers showed no effect on cancer incidence, and studies with PCSK9 inhibition and statins also did not show any association between cancer and PCSK9 (Folsom et al., 2007; Yarmolinsky et al., 2020). Having said this we have to mention that experimental studies suggest that PCSK9 protects cancer cells against apoptosis. Although it is currently unclear whether this will lead to new therapeutic options against specific types of cancer in future, it is clear that it is mechanistically very interesting, showing quite different roles for PCSK9 in different organs. In BON-1 cells, a frequently used model system of pancreatic neuroendocrine tumor (PNET) research, silencing PCSK9 expression by either agomirs of miR-224 or by siRNA directed against PCSK9 increased the rate of apoptosis (Bai et al., 2016). Similarly, silencing PCSK9 in human lung adenocarcinoma cells (A549 cells) increased apoptosis as characterized by bcl-2/bax modulation, caspase activation, and the downregulation of survivin and the X-linked inhibitor of apoptosis protein (Xu et al., 2017). These examples suggest that PCSK9 can protect cells against apoptosis making them more resistant against cancer therapy. In contrast to the lack of general association between PCSK9 and cancer, in the context of breast cancer, there is an association between loss-of-function carriers and gain-of-function carriers and breast cancer. In these cells, silencing PCSK9 expression, linked to the upregulation of LDL-R, is associated with increased survival (Momtazi-Borojeni et al., 2019; Abdelwahed et al., 2020). Similarly, PCSK9 is strongly expressed in and secreted by gastric tumors (Marimuthu et al., 2013). Collectively these data suggest that in cancers that benefit from high LDL-C, PCSK9 improves cancer progression, whereas in cells in which PCSK9 protects against apoptosis it makes cells more resistant against therapy. However, epidemiological data do not support a strong correlation between cancer and PCSK9 in general.

## Discussion

Basic research has indicated that PCSK9 is a main controller of metabolic fine tuning in almost all organs but specifically in those that dominate the regulation of metabolism (Table 2). It is important to recognize that PCSK9 is required for metabolic homeostasis as indicated by long-term alterations in PCSK9^−/−^ mice under basal and stress conditions. Furthermore, data in exercise models identify non-harmful upregulation of PCSK9 as well. Finally, the cross-activation of the hepatic expression of PCSK9 by stress in the heart and kidney indicates the importance of PCSK9 for metabolic adaptation between different organs. These regulatory roles are immediately affected under conditions such as inflammation. Whether under such conditions, the induction is simply too strong or whether the co-activation of pro-inflammatory pathways in parallel shifts these initially meaningful adaptations into maladaptation is not fully understood. In the clinical context of hypercholesterolemia and PCSK9 treatment, it is clear that most patients will benefit from reducing PCSK9 levels because under such conditions, the level of PCSK9 is far too high. However, a better understanding of the local and non-hepatic effects of PCSK9 and the identification of potential target proteins in non-hepatic tissues will definitively improve our understanding of the regulation of metabolism as well as that of potential therapeutic options.

## Author Contributions

K-DS: manuscript preparation. AW and RS: manuscript editing. All authors contributed to the article and approved the submitted version.

### Conflict of Interest

The authors declare that the research was conducted in the absence of any commercial or financial relationships that could be construed as a potential conflict of interest.
